# Immune Response in Critically Ill Patients

**DOI:** 10.1155/2018/9524315

**Published:** 2018-07-16

**Authors:** Maja Surbatovic, Danilo Vojvodic, Wasim Khan

**Affiliations:** ^1^Clinic of Anesthesiology and Intensive Therapy, Military Medical Academy, Crnotravska 17, 11000 Belgrade, Serbia; ^2^Faculty of Medicine of the Military Medical Academy, University of Defence, Crnotravska 17, 11000 Belgrade, Serbia; ^3^Institute for Medical Research, Military Medical Academy, Crnotravska 17, 11000 Belgrade, Serbia; ^4^Division of Trauma & Orthopaedic Surgery, Addenbrooke's Hospital, University of Cambridge, Cambridge CB2 2QQ, UK

Critical illness is defined by presence of altered organ function in acutely ill patients such that homeostasis cannot be maintained without medical intervention in intensive care units, such as mechanical ventilation, vasoactive support for hemodynamics, renal replacement therapy, and so on. It usually involves two or more organ systems. Immune dysfunction is common in critically ill patients and it may modulate immune response and affect patient morbidity and mortality, particularly in severe trauma and/or sepsis. Immune cells and mediators, in critical care setting, are understudied and do represent challenging area. Better understanding of potential beneficial and/or harmful effects of pro- and/or anti-inflammatory response will improve process of intensive care. Potential therapeutic interventions may improve clinical outcome. Pro- and anti-inflammatory mediators can be predictive biomarkers of organ dysfunction and outcome in critically ill patients also [1]. Immune cells' and mediators' role in immune response in critical illness is not yet fully elucidated. Also, their role as predictive biomarkers of organ dysfunction and outcome is of interest for both researchers and clinicians. Profound knowledge of this complex conundrum is essential for potential therapeutic procedures regarding immune dysfunction in critically ill patients.

Source of sepsis is important component influencing both biomarkers in systemic circulation and therapeutic options regarding source control [2]. In this special issue, M. Holub et al. focused their research on correlation of selected biomarkers with origin and severity of sepsis. They analyzed the plasma levels of cytokines (IL-6, IL-8, and IL-10), chemokines (CCL2/MCP-1, MIP-1*β*), heparin-binding protein (HBP), soluble CD14 (sCD14), and cortisol in the blood from septic patients obtained during the first 96 hours of intensive care unit hospitalization. The authors showed that plasma concentrations of MCP-1, sCD14, IL-6, and IL-10 were significantly higher in patients with community-acquired pneumonia and infective endocarditis compared to those with bacterial meningitis. Their findings suggest that MCP-1, sCD14, IL-6, IL-10, cortisol, and HBP are modulated by the source of sepsis and that elevated MCP-1 and cortisol plasma levels are associated with sepsis-induced organ dysfunction.

The cholinergic system plays an important role in maintaining and modulating an adequate immune response upon an inflammatory episode. Cholinergic activity, acting extrasynaptically (nonneuronal acetylcholine), has been proposed to be a major mediator of the neuroimmune response to inflammation in a classical feedback reflex manner [3]. Serum cholinesterase (BChE), an enzyme that hydrolyzes acetylcholine, plays an important role during inflammatory response and serves as an accurate index of cholinergic activity. In this special issue, A. Zivkovic et al. investigated BChE activity in septic patients using a point-of-care system. Levels of conventional inflammatory markers (C-reactive protein, procalcitonin, IL-4, IL-6, IL-10, TNF-*α*, and midrange-proadrenomedullin—MR-proADM) were also measured. The authors observed a strong, sustained reduction in BChE activity in nonsurvivors as compared to survivors in a 90-day observation period. Reduced BChE activity when measured at the ICU admission effectively differentiated between the 90-day survivor and the nonsurvivor patient groups. They estimated a critical BChE level of 1.661 kU/l (CI 0.5–0.8, 94% sensitivity, 48% specificity, and AUC 0.7) to best predict patient outcome.

It is difficult to find adequate biomarker of immune response in critical illness, regardless of its cause, with good predictive value regarding outcome because there is wide and complex array of immune-related mediators. Many of them were explored in this clinical setting with contradictory results. Recently, some readily available parameters, originated from routine complete blood count (CBC), have been investigated as potential biomarkers [4, 5]. In this special issue, Surbatovic et al. assessed prognostic value of neutrophil-to-lymphocyte ratio (NLR), monocyte-to-lymphocyte ratio (MLR), platelet-to-lymphocyte ratio (PLR), and mean platelet volume-to-platelet count (MPV/PC) ratio regarding outcome in total of 392 critically ill patients with secondary sepsis and/or trauma. Patients were divided into four subgroups: peritonitis, pancreatitis, trauma with sepsis, and trauma without sepsis. The authors demonstrated that NLR and MPV/PC levels were significantly higher in nonsurvivors (AUC/ROC of 0.681 and 0.592, respectively, in peritonitis subgroup; 0.717 and 0.753, respectively, in pancreatitis subgroup); MLR and PLR did not differ significantly. There was no significant difference of investigated biomarkers between survivors and nonsurvivors in trauma patients with and without sepsis except for PLR in trauma without sepsis subgroup (significantly higher in nonsurvivors; AUC/ROC of 0.719). Independent predictor of lethal outcome was NLR in whole cohort and in peritonitis subgroup as well as MPV in pancreatitis subgroup. Also, there were statistically significant differences in MPV/PC, MLR, and PLR values regarding nature of bacteremia. In general, the lowest levels had patients with Gram-positive blood cultures. The authors concluded that NLR and MPV were very good independent predictors of lethal outcome. For the first time, they demonstrated that nature of bacteremia influences MPV/PC, MLR, and PLR.

Neutrophils are one of the first lines of defense against invading pathogens and are responsible for containing and eliminating invading pathogens. Neutrophils are multifaceted innate immune cells that also modulate the inflammatory response and initiate the adaptive immune responses to sepsis via the release of cytokines [6]. Following activation, triggered either by frustrated phagocytosis or sustained inflammation, neutrophils release neutrophil extracellular traps (NETs), whereby nuclear DNA laden with histones and granular contents are liberated into the extracellular space, which trap and kill extracellular bacteria [7]. In this special issue, J. Patel et al. presented a prospective observational cohort study enrolling 21 healthy age-matched controls and 39 sepsis and 60 severe sepsis patients from acute admissions to two UK hospitals. Patients had sequential bloods for the ex vivo assessment of NETosis in response to phorbol myristate acetate (PMA) using a fluorometric technique and chemotaxis using time-lapse video microscopy. The authors demonstrated that ex vivo NETosis was significantly reduced in patients with severe sepsis, compared to patients with sepsis and controls. PMA NETosis from patients with septic shock was significantly reduced further compared to controls. Reduced NETosis at baseline was significantly associated with an increased 30-day and 90-day mortality in sepsis patients. These findings were accompanied by defects in neutrophil migration and delayed apoptosis. Resolution of sepsis was not associated with the return to baseline levels of NETosis or migration. The authors concluded that sepsis induces significant changes in neutrophil function with the degree of dysfunction corresponding to the severity of the septic insult which persists beyond physiological recovery from sepsis. The changes induced lead to the failure to effectively contain and eliminate the invading pathogens and contribute to sepsis-induced immunosuppression. For the first time, they demonstrated that reduced ex vivo NETosis is associated with poorer outcomes from sepsis.

Sepsis progression and septic liver dysfunction are characterized by complex pathophysiological alterations, including processes like reactive oxygen species (ROS) and nitrogen species (RNS) release, inflammation, and apoptosis [8]. In this special issue, Z. Jiang et al. used a lipopolysaccharide (LPS) stimulation model to simulate the septic liver injury and to investigate the effect of Sophora alkaloid sophocarpine on LPS-stimulated mice with endotoxemia. The authors demonstrated that sophocarpine increases the survival rate of mice and attenuates the LPS-induced liver injury, which is indicated by pathology and serum liver enzymes. Further research found that sophocarpine ameliorated hepatic oxidative stress indicators (H_2_O_2_, O_2_^−^, and NO) and enhanced the expression of antioxidant molecules such as superoxide dismutase (SOD), catalase (CAT), and glutathione (GSH). In addition, sophocarpine also attenuated regional and systematic inflammation and further reduced apoptosis of hepatocytes. Mechanistic evidence was also investigated in this study as sophocarpine inhibited hepatic expression of the CYP2E/Nrf2 pathway during oxidative stress, inactivated p38/JNK cascade and NF-*κ*B pathway, and suppressed PI3K/AKT signaling that reduced apoptosis. The authors concluded that their investigation unveiled the protective role of sophocarpine in LPS-stimulated oxidative reaction, inflammation, and apoptosis suggesting its promising role in attenuating inflammation and liver injury in septic endotoxemia.

Trauma has profound effect on immune response and may lead to diminished functionality of monocytes and subsequently their IL-1*β* release. IL-1*β* plays an important role in host immunity and protection against infections. Its biological activation via IL-1*β* precursor processing requires the transcription of inflammasome components and their activation. Activity of NOD-like receptor inflammasomes- (NLR-) like NLRP3 leads to the maturation of IL-1*β* [9]. In this special issue, S. Kany et al. investigated the role of the NLRP3 inflammasome in impaired monocyte functionality after a traumatic injury. Ex vivo-in vitro stimulation of isolated CD14^+^ monocytes with lipopolysaccharide (LPS) showed a significantly higher IL-1*β* secretion in healthy volunteers compared to trauma patients after admission. Reduced IL-1*β* secretion was paralleled by significantly lowered gene expression of NLRP3 in monocytes from trauma patients compared to those of healthy volunteers. Transfection of monocytes with NLRP3-encoding plasmid recovered the functionality of monocytes from trauma patients regarding the IL-1*β* secretion. The authors concluded that CD14^+^ monocytes from trauma patients are significantly diminished in their function, and that the presence of NLRP3 components is necessary in recovering the ability of monocytes to produce active IL-1*β*. Also, this recovery of the NLRP3 inflammasome in monocytes may imply a new target for treatment and therapy of immune suppression after severe trauma.

Finally, immune response is important component in very specific setting of out-of-hospital cardiac arrest (OHCA). Survival of OHCA is affected by global ischaemia followed by a pronounced reperfusion injury due to return of spontaneous circulation (ROSC). The ischaemia-reperfusion response induces a complex activation of immunologic pathways leading to subsequent systemic inflammatory immune reaction. This phenomenon is known as the postcardiac arrest syndrome (PCAS). Induction of therapeutic hypothermia (TH) in this setting also influences immunoinflammatory response [10]. In this special issue, M. Braunstein et al. evaluated immediate immunological changes following cardiopulmonary resuscitation (CPR). mRNA expression levels of selected immunomodulatory cytokines in OHCA survivors were detected and correlated to clinical parameters. OHCA survivors with sustained unconsciousness after return of spontaneous circulation (ROSC) were included. PAXgene whole blood samples were drawn immediately after initiation of CPR and subsequently after 6 h, 12 h, 24 h, 48 h, and 72 h. TNF-alpha, IL-8, IL-10, and IL-1ra mRNA levels were quantified by RT-qPCR and compared to multiple organ failure, a 30-day survival, and the induction of TH. The authors demonstrated characteristic time-dependent cytokine profile in the early postresuscitation period. High initial TNF-alpha and IL-8 mRNA levels were followed by a significant decrease. IL-1ra mRNA levels significantly increased beginning after 6 h. Nonsurvivors showed significantly higher IL-8 mRNA levels immediately after CPR. TH induced significantly higher IL-1ra mRNA levels compared to normothermia. The authors concluded that significant mRNA cytokine expression changes are already detectable immediately after initiation of CPR. These expressional changes are significantly different depending on a 30-day survival. Also, TH seems to attenuate proinflammatory immune reaction by a significant increase of IL-1ra mRNA levels.

Current special issue provides broad spectrum of interesting topics in the field of immune response in critically ill. Hopefully, it will be useful to both clinicians and researchers in this exciting field.

*Maja Surbatovic* *Danilo Vojvodic* *Wasim Khan*
